# Retrospective Study of Factors Affecting the Accuracy of Predicting Vancomycin Concentrations in Patients Aged 75 Years and Above

**DOI:** 10.3390/medicina60081273

**Published:** 2024-08-07

**Authors:** Masaki Takigawa, Hiroyuki Tanaka, Masako Kinoshita, Toshihiro Ishii, Masayuki Masuda

**Affiliations:** 1 Department of Clinical Pharmaceutics, Faculty of Pharmaceutical Sciences, Toho University, Funabashi 274-8510, Japan; 2 Department of Practical Pharmacy, Faculty of Pharmaceutical Sciences, Toho University, Funabashi 274-8510, Japan

**Keywords:** Bland–Altman plot, creatinine clearance, older people, predictive performance, therapeutic drug monitoring, vancomycin

## Abstract

*Background and Objectives*: The predicted serum concentrations of vancomycin are determined using population pharmacokinetic parameters. However, the accuracy of predicting vancomycin serum concentrations in the older population remains unclear. Therefore, this study aimed to investigate the accuracy of predicting vancomycin serum concentrations and identifying elements that diminish the prediction accuracy in older people. *Materials and Methods*: A total of 144 patients aged 75 years or older were included. The serum vancomycin concentrations in the patients were predicted based on population pharmacokinetic parameters common in Japan. We examined the accuracy of serum vancomycin concentration prediction in elderly individuals by comparing the predicted and measured serum vancomycin concentrations in each patient. The prediction accuracy was evaluated using the mean prediction error (ME) and mean absolute error of prediction (MAE) calculated from the measured and predicted serum vancomycin concentrations in each patient. *Results*: The ME for all patients was 0.27, and the 95% CI included 0, indicating that the predicted values were not significantly biased compared to the measured values. However, the predicted serum concentrations in the <50 kg body weight and serum creatinine (Scr) < 0.6 mg/dL groups were significantly biased compared to the measured values. The group with a history of intensive care unit (ICU) admission showed the largest values for the ME and MAE. *Conclusions*: Our prediction accuracy was satisfactory but tended to be lower in underweight patients, those with low creatinine levels, and patients admitted to the ICU. Patients with multiple of these factors may experience a greater degree of decreased predictive accuracy.

## 1. Introduction

Vancomycin, a glycopeptide antimicrobial agent, is active against a variety of Gram-positive cocci and is frequently used in the treatment of methicillin-resistant *Staphylococcus aureus* and methicillin-resistant *Staphylococcus epidermidis* in infectious disease treatment settings [1]. Therapeutic drug monitoring (TDM) is necessary for ensuring the efficacy and safety of vancomycin administration [2]. In recent years, area under the curve (AUC)-based dosing has replaced trough-based dosing and has become the recommended TDM for vancomycin [2,3], with a shift from targeting a trough value of 10–15 mg/dL to targeting an AUC of 400–600 μg-h/mL.

Vancomycin-associated nephropathy is a typical adverse event of vancomycin use. Various risk factors are associated with the development of vancomycin-associated nephropathy [4]; aging [5,6] has been reported as one of the risk factors. Furthermore, the serum vancomycin concentration is associated with the development of vancomycin-associated nephropathy in elderly individuals [7,8]. Thus, elderly patients require a careful dosing design because high serum vancomycin concentrations cause vancomycin-associated nephropathy. When designing vancomycin dosing, PPK parameters are used to predict serum vancomycin concentrations. In Japan, vancomycin dosing design is based on the PPK parameters reported by Yasuhara et al. [9] and Yamamoto et al. [10]. The PPK-based TDM software (SHIONOGI-VCM-TDM ver.2009) by Yasuhara et al. rounds up the serum creatinine level to 0.6 mg/dL when it is less than 0.6 mg/dL. However, it has been reported that this method may overestimate renal function in elderly patients with a creatinine clearance (Ccr) > 85 mL/min [11]. In the PPK-based software (Vancomycin MEEK TDM analysis software Ver3.3) by Yamamoto et al., Ccr is constant in patients with Ccr > 85 mL/min. This is based on the fact that Ccr does not correlate with vancomycin clearance and is constant in the group with Ccr > 85 mL/min. However, it has been reported that the Ccr and vancomycin clearance may correlate even when Ccr is >85 mL/min in younger men [11]. Currently, in Japan, the design of AUC-guided dosing using the PPK parameters reported by Yasuhara et al. is recommended. When determining vancomycin dosing, Ccr is used to assess the renal function and calculate the predicted serum concentrations. The serum creatinine (Scr) levels are used to calculate Ccr, but accurate Ccr determination in the older population is challenging due to potential reductions in muscle mass. Consequently, caution is advised when predicting serum concentrations in the older population. Previous reports have highlighted the utility of correcting for Scr [12] and devising renal function assessment [13] to improve the accuracy of predicting vancomycin serum concentrations in older patients. Furthermore, in our previous study [14], we investigated the predictive accuracy of using various renal function parameters when predicting serum concentrations of vancomycin using the PPK parameters of Yamamoto et al. The results showed that the use of the estimated glomerular filtration rate (eGFR) determined using the Berlin Initiative study-1 equation for renal function assessment may improve the accuracy of predicting vancomycin concentrations in elderly patients compared to the use of Ccr. However, this result was not a significant enough improvement in the prediction accuracy to be clinically applicable. In addition, we were unable to examine the differences in the prediction accuracy due to differences in the patients’ backgrounds.

As mentioned earlier, various efforts have been made to improve the accuracy of predicting the serum concentrations of vancomycin in older adults. However, there are no reports that have investigated in detail the accuracy of predicting vancomycin serum concentrations in different backgrounds of the older population. Furthermore, there is a lack of investigation into factors that may reduce the accuracy of these predictions. Therefore, the purpose of this study was to investigate the accuracy of predicting vancomycin serum concentrations based on the diverse backgrounds of older patients aged 75 years or older.

## 2. Materials and Methods

### 2.1. Patients and Survey Items

In this study, the older population was defined as individuals aged 75 years or older. We conducted a retrospective study on patients in this age group who received vancomycin from January 2019 to December 2021 at the Tokyo Metropolitan Institute for Geriatrics and Gerontology. This study involved patients who had their serum vancomycin concentrations measured, regardless of the type of infection. Patients were excluded based on the following criteria: (i) those with an administration period of 3 days or less; (ii) patients who died during the administration period or within 30 days after the end of administration to eliminate cases associated with multiple organ failure; (iii) individuals undergoing hemodialysis; (iv) patients who developed acute kidney injury (AKI) during the administration period (AKI was defined as a rise in the Scr level of at least 0.3 mg/dL (or at least 50%) concerning the kidney improving global outcomes criteria [15]); or (v) patients with no vancomycin serum concentrations measured. We collected electronic records, including patient data (age, sex, body weight, body mass index [BMI], albumin (alb), aspartate aminotransferase, alanine transaminase, Scr, blood urea nitrogen [BUN], and vancomycin data (vancomycin dosage and measurement of the initial vancomycin trough concentrations). Vancomycin serum concentrations were measured using a homogeneous enzyme immunoassay at the Tokyo Metropolitan Institute for Geriatrics and Gerontology laboratory. Ccr was calculated from the obtained data using the Cockcroft and Gault equation. The Cockcroft and Gault equation is shown below. If Scr was less than 0.6, Scr was rounded up to 0.6.

Ccr (mL/min) = (140 − Age) × Body weight/(72 × Scr) × (0.85 if female)(1)

### 2.2. Calculation of Predicted Serum Vancomycin Concentrations

We calculated the predicted serum vancomycin concentrations using the Towa-TDM analysis software Ver3.3 (Towa Pharma International Holdings Co., Ltd., Osaka, Japan). This software is based on population parameters reported by Yasuhara et al. [9]. The PPK parameters reported by Yasuhara et al. are shown below.

CL (L/h) = 0.797 × Ccr (L/h)(2)

The volume of distribution at steady state (Vdss) (L) = 60.7(3)

K_12_ (h^−1^) = 0.525 (4)

K_21_ (h^−1^) = 0.213 (5)

K_12_ is the transfer rate constant from the central compartment to the peripheral compartment, and K_21_ is the transfer rate constant from the peripheral compartment to the central compartment. The PPK parameters of Yasuhara et al. [9] was generated with data on 190 participants (131 male and 59 female individuals) with methicillin-resistant *Staphylococcus aureus* infection. The mean (range) age of the participants was 64.3 (19.3–89.6) years and Scr was 1.21 (0.20–13.4) mg/dL. Mean Ccr was 77.1 mL/min with a minimum of 6.85 mL/min.

### 2.3. Evaluation of the Agreement between Measured and Predicted Serum Vancomycin Concentrations

The Bland–Altman plot was generated to visually identify the discrepancy between the measured and predicted serum concentrations of vancomycin. The limits of agreement (LOA) were also calculated.

### 2.4. Evaluation of the Serum Vancomycin Concentration Prediction

For the assessment of predictive precision, we computed the mean prediction error (ME) using equation 6 and the mean absolute prediction error (MAE) using Equation (7); ME served as an indicator of predictive bias, and MAE served as an indicator of predictive accuracy.

(6) $$\mathrm{ME}=\sum_{i=1}^{n}(C\mathrm{prediction}-C\mathrm{measured})/n$$

(7) $$\mathrm{MAE}=\sum_{i=1}^{n}|C\mathrm{prediction}-C\mathrm{measured}|/n$$

C*_prediction_* is the predicted serum concentration calculated by the PPK model of Yasuhara et al., and C*_measured_* is the measured value. Smaller values of ME and MAE indicated lower bias and increased accuracy. To assess significant bias between predicted and measured values, the 95% confidence interval (CI) of ME was computed using Student’s t distribution. Subsequently, the confirmation of whether the 95% confidence interval (CI) encompassed 0 was conducted. ME and MAE were listed and compared for all eligible patients and by patient background (sex, body weight less than or greater than 50 kg, alb less than or greater than 2.5 g/dL, Scr less than or greater than 0.6 mg/dL, and Ccr less than or greater than 30 mL/min). Concomitant use of piperacillin/tazobactam may potentially increase Scr [16], while ICU residents with unstable body circulation [17] pose challenges in predicting vancomycin serum concentrations. We speculated that these two factors might also affect the accuracy of predicting serum vancomycin concentrations; thus, we investigated the ME and MAE.

### 2.5. Statistical Analysis

Continuous data are presented as median (range). ME and MAE are presented as 95% CI. Statistical analyses were performed using JMP Pro 17 (SAS Institute Inc., Cary, NC, USA).

### 2.6. Ethical Approval

As this was a retrospective study, written informed consent was not obtained from the study participants. However, all patients gave prior consent for the use of their samples and other materials generated during the course of their medical care for future research. Furthermore, the patients were provided an opt-out opportunity. This study was approved by the ethics committee of the Tokyo Metropolitan Institute for Geriatrics and Gerontology (Approval No. R21-105).

## 3. Results

### 3.1. Patients

**Figure 1:**
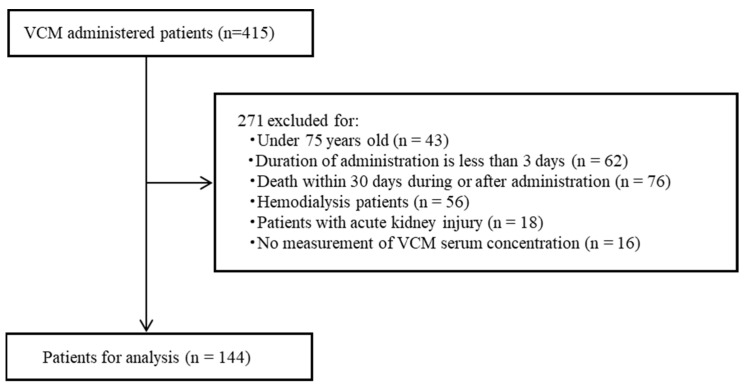
Selection of patients for this study.

A total of 415 patients were administered vancomycin during the study period. This study excluded 271 patients after applying the exclusion criteria, and 144 patients were included in the analysis (Figure 1). The primary reason for exclusion was death during or 30 days after vancomycin administration (*n* = 76). Table 1 summarizes the patient characteristics. The median age was 84.5 years, and the median vancomycin serum concentration was 9.5 μg/mL. The most prevalent site of infection in the target patients was the respiratory tract, which was observed in 50 patients. The second most common site of infection was the catheter, which was observed in 19 patients.

### 3.2. Agreement between the Predicted and Measured Vancomycin Serum Concentrations

**Figure 2:**
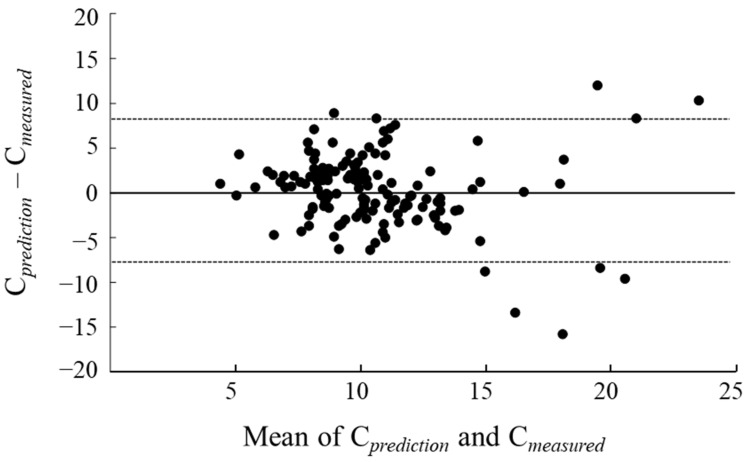
Bland–Altman plot of measured and predicted serum vancomycin concentrations. The dashed lines denote the 95% limits of agreement. C*_measured_*, measured vancomycin serum concentration; C*_prediction_*, predicted vancomycin serum concentration.

The Bland–Altman plot is illustrated in Figure 2. The upper and lower LOA were 8.2 and −7.7, respectively. Many plots were within the LOA, whereas some plots exceeded it when the average C*_prediction_* and C*_measured_* were greater than 15 µg/dL.

### 3.3. Predictive Accuracy and Precision of the Serum Vancomycin Concentrations

Table 2 shows the values for ME and MAE. The ME value for all the patients was 0.27, and the 95% CI included 0; thus, the predicted value was not significantly biased compared with the measured value. The ME for the body weight < 50 kg group was 0.96, for the Alb > 2.5 g/dL group was 0.97, and for the Scr < 0.6 mg/dL group was 1.37, none of which included 0 in the 95% CI; thus, the predicted serum concentrations were significantly biased compared to the measured values. The predicted serum concentrations tended to be larger than the measured values because of the positive ME values in the body weight < 50 kg and Scr < 0.6 mg/dL groups. The ME for the group with a history of ICU admission was the largest at 3.13, although the 95% CI included 0, indicating that it was not significantly biased. The MAE across patient backgrounds was generally similar in each group, with the largest MAE observed in the group with a history of ICU admission at 4.17.

## 4. Discussion

In this study, we assessed the prediction accuracy of vancomycin serum concentrations in patients aged 75 years or older, considering various patient backgrounds, by calculating the ME and MAE. The prediction accuracy of the predicted serum concentrations calculated using PPK, a common practice in Japan, was generally satisfactory. The calculated ME results showed a significant bias toward the measured values in the body weight < 50 kg and Scr < 0.6 mg/dL groups. Although there was considerable bias in the ICU admissions, it did not reach statistical significance. Notably, the calculated MAE values were the highest among the patients with a history of ICU admission. This study is the first to investigate the accuracy of predicting the serum concentrations of vancomycin in older patients with different backgrounds.

The overall ME for the patients in this study was 0.27 (95% CI: −0.40–0.94), indicating no significant bias, as the 95% CI included 0. The MAE value was 3.02. In a study including younger patients, Tanaka et al. [18] examined the predictive accuracy of serum vancomycin concentrations in a patient population averaging 68 years of age. Tanaka et al. [18] calculated the predicted serum concentrations using the same method as ours and reported a significant bias of −1.88 (95% CI: −2.84–−0.92) for the ME and an MAE value of 4.42. Comparing the ME values in this study with those reported by Tanaka et al., the ME values were smaller and not significantly biased, and the MAE values were also smaller. Bland–Altman plots were generated to investigate the agreement between the predicted and measured serum concentrations, and many cases were within the LOA range. These results suggest that the prediction accuracy of the vancomycin serum concentrations in older patients is generally good when the predicted serum concentrations are calculated using PPK by Yasuhara et al. However, in the Bland–Altman plot, the measured value and predicted value did not agree in some cases when the vancomycin serum concentrations were high. Therefore, caution should be exercised if the vancomycin serum concentrations are maintained at high levels. We have previously investigated the accuracy of predicting vancomycin serum concentrations in a population of patients aged 75 years and older, but the ME was 3.70 (95% CI: 2.73–4.66), which differs from the results of our current study and is significantly biased [14]. The major difference between the results of this study and those of our previous reports is the method used to calculate the predicted serum concentrations of vancomycin. In our previous reports, the predicted blood concentrations were calculated using the PPK reported by Yamamoto et al. [10]. Imai et al. [11] studied the difference in predicted serum concentrations when using the PPK from Yasuhara et al. and Yamamoto et al. Imai et al. found that in patients aged 65 years and older, the ME was −0.91 (95%CI: −2.11–0.32) when dose design was performed with PPK-based software by Yasuhara et al. and 3.67 (95%CI: 2.54–4.80) when dose design was performed with PPK-based software by Yamamoto et al. Thus, the ME value was smaller and not significantly biased toward the measured value when the dosage design was based on the PPK of Yasuhara et al. Imai et al. attributed this to the differences in the clearance parameters of both PPKs. The same reason may account for the difference between the results of this study and those of our previous reports. The PPK of Yasuhara et al. may be more suitable for calculating the predicted serum concentrations of vancomycin in the older population. In our previous study [14], we found that the accuracy of predicting serum vancomycin concentrations may be improved when the renal function is assessed using the eGFR calculated with the Berlin Initiative Study-1 equation. The use of the eGFR based on the Berlin Initiative Study-1 equation might have also improved the prediction accuracy in this study. However, the current vancomycin dosing design used in Japan uses Ccr to assess renal function. Therefore, Ccr was used to assess the renal function in this study.

When investigating the prediction accuracy based on the background of the patients, the MEs for the body weight < 50 kg and Scr < 0.6 mg/dL groups did not include 0 in the 95% CI, and the predicted serum concentrations were significantly biased toward the measured values. Although the group with a history of ICU admission had the highest ME value of 3.13, the actual value was not significantly biased. Similarly, the MAE value was the highest at 4.17. Patients who are lean [19] or hemodynamically unstable [17] have been reported as a group of patients for whom serum concentrations are difficult to predict. The results of this study suggest that the prediction accuracy of vancomycin serum concentrations may be less reliable in older patients who are lean or hemodynamically unstable. In this study, for patients in the Scr < 0.6 mg/mL group, the predicted serum concentrations were calculated by rounding up to Scr = 0.6 mg/mL. When designing vancomycin dosing, some reports [20] recommend rounding up to Scr = 0.6 mg/mL in patients with Scr < 0.6 mg/mL. However, our findings indicate that this practice may not consistently yield favorable outcomes. The ME values in the Alb ≥ 2.5 g/dL group were also significantly biased. Positive ME values tended to be lower for the actual values than for the predicted values. It has been reported that low Alb levels increase free vancomycin concentrations and enhance clearance, resulting in low serum vancomycin concentrations [21]. On the contrary, it has also been reported that the Alb level is not a variable factor in vancomycin concentrations [22]. These facts are not in agreement with the results of the present study. In the present study, only an Alb level of 2.5 g/dL was used as the cutoff value. We believe that it is necessary to re-examine the findings of this study in the future by setting multiple cutoff values for the Alb level. The MAE values in the Ccr < 30 mL/min group ranked second only to those with a history of ICU admission. Previous reports [23] have similarly highlighted the challenges in predicting serum concentrations in patients with renal impairment, suggesting that this difficulty extends to older populations. Additionally, studies involving patients over 80 years of age [24] have found that Ccr and body weight affect the PPK of vancomycin, aligning with the results of this study. In critical care settings, augmented renal clearance (ARC) can pose a challenge [25]. Increased renal clearance due to ARC may result in insufficient doses of renally excreted drugs [26]. The results of this study also showed a positive ME of 3.13 for patients with ICU admissions, indicating that the actual measured value was lower than the predicted serum concentrations. This discrepancy may be attributed to the presence of ARCs. This finding emphasizes the importance of recognizing the potential for dose shortages due to ARC by healthcare practitioners, even within the older population, particularly in critical care settings. Chu et al. [27] reported a PPK-based model for patients with ARC. In addition, Zhou et al. [28] presented an individualized vancomycin administration model for older adult patients with sepsis, potentially facilitating tailored dosing for this population. These PPK models may improve the accuracy of predicting vancomycin concentrations in patients admitted to the ICUs.

The present study had some limitations. First, this was a retrospective study conducted at a single institution, and the possibility of patient bias cannot be ruled out. Second, in the subgroup of patients identified with a low prediction accuracy in this study, the discrepancy between the predicted and actual blood serum concentrations may not consistently represent a clinically meaningful difference. Third, we were not able to investigate the prediction accuracy for patients with diverse backgrounds, a factor addressed in this study. Finally, the TDM of vancomycin is currently dominated by AUC-based dosing, although TDM by trough-based dosing is also used in some cases [29]. Further research is required to determine the applicability of the study results to AUC-based dosing scenarios. In this study, trough values, which are easily assessable, were used for the evaluation.

## 5. Conclusions

In this study, we investigated the accuracy of PPK for predicting vancomycin serum concentrations in persons aged 75 years or older using the PPK model reported by Yasuhara et al. The results suggested that the prediction accuracy was satisfactory, although it tended to be diminished in underweight patients, patients with low creatinine levels, and those admitted to the ICU. In patients with these factors, the degree of decreased predictive accuracy was small. However, patients with multiple factors may have a greater degree of decreased predictive accuracy. Currently, the TDM method used for vancomycin is AUC-based dosing. Since the PPK by Yasuhara et al. is used to calculate the predictive AUC in Japan, we believe that the results of this study may be helpful.

## Figures and Tables

**Table 1:** Baseline characteristics of the patients.

Characteristics	Median (Range) or No. of Patients (%)	
Age	84.5	(75–99)
Sex		
Male	80	(55.6)
Female	64	(44.4)
Body weight (kg)	46.8	(26.0–78.2)
BMI	19.4	(11.0–32.0)
Vancomycin dose (mg/kg/day)	16.9	(3.4–43.2)
Alb (g/dL)	2.4	(1.2–4.5)
AST (U/L)	27.0	(7.0–328.0)
ALT (U/L)	20.0	(3.0–197.0)
Scr (mg/dL)	0.8	(0.16–4.91)
BUN (mg/dL)	19.0	(5.0–119.0)
Ccr (mL/min)	42.2	(6.6–134.5)
Vancomycin serum concentration (μg/mL)	9.5	(3.0–26.0)
Infection site		
Respiratory tract	50	(34.7)
Catheter	19	(13.2)
Sepsis and bacteremia	16	(11.1)
Skin and soft tissue	15	(10.4)
Liver and biliary tract	8	(5.6)
Urinary tract	6	(4.2)
Muscle and bone	6	(4.2)
Febrile neutropenia	3	(2.1)
central nervous system	2	(1.4)
Surgical Site Infection	2	(1.4)
Others/Unknown	17	(11.8)

BMI, body mass index; Alb, albumin; AST, aspartate aminotransferase; ALT, alanine aminotransferase; Scr, serum creatinine; BUN, blood urea nitrogen; Ccr, creatinine clearance calculated using the Cockcroft–Gault equation.

**Table 2:** Comparison of prediction accuracy by patient characteristics.

Characteristics		ME (95% CI)	MAE (95% CI)
Total (*n* = 144)		0.27 (−0.40, 0.94)	3.02 (2.57, 3.47)
Sex	Male (*n* = 80)	0.55 (−0.32, 1.41)	2.93 (2.34, 3.51)
	Female (*n* = 64)	−0.08 (−1.15, 0.98)	3.14 (2.43, 3.85)
Body weight (kg)	<50 (*n* = 86)	0.96 (0.18, 1.74)	2.76 (2.22, 3.30)
	≧50 (*n* = 58)	−0.76 (−1.93, 0.42)	3.41 (2.63, 4.18)
Piperacillin/tazobactam combination	With (*n* = 18)	1.03 (−0.69, 2.75)	2.86 (1.80, 3.91)
	Without (*n* = 126)	0.16 (−0.57, 0.89)	3.04 (2.55, 3.53)
Alb (g/dL)	<2.5 (*n* = 77)	−0.34 (−1.34, 0.66)	3.18 (2.49, 3.87)
	≧2.5 (*n* = 67)	0.97 (0.10, 1.83)	2.84 (2.28, 3.40)
Scr (mg/dL)	<0.6 (*n* = 40)	1.37 (0.39, 2.34)	2.52 (1.83, 3.21)
	≧0.6 (*n* = 104)	−0.16 (−1.00, 0.69)	3.21 (2.65, 3.78)
Ccr (mL/min)	<30 (*n* = 35)	0.22 (−1.40, 1.84)	3.76 (2.81, 4.71)
	≧30 (*n* = 109)	0.28 (−0.45, 1.01)	2.78 (2.28, 3.29)
ICU admission	With (*n* = 11)	3.13 (−0.11, 6.38)	4.17 (1.57, 6.77)
	Without (*n* = 133)	0.03 (−0.64, 0.70)	2.93 (2.48, 3.37)

BMI, body mass index; Alb, albumin; Scr, serum creatinine; Ccr, creatinine clearance calculated using the Cockcroft and Gault equation; ICU, intensive care unit.

## Data Availability

The original contributions presented in this study are included in the article; further inquiries can be directed to the corresponding author.
